# Molecular analysis of feces reveals gastrointestinal nematodes in reintroduced wild asses of the Negev desert

**DOI:** 10.1016/j.ijppaw.2024.100980

**Published:** 2024-08-29

**Authors:** R. Forman, M. Lalzar, M. Inbar, T.S. Berman

**Affiliations:** a Department of Evolutionary and Environmental Biology, University of Haifa, Israel; b Bioinformatics Services Unit, University of Haifa, Israel; c Hula Research Centre, Department of Animal Sciences, Tel-Hai Academic College, Upper Galilee, 1220800, Israel; d MIGAL- Galilee Research Institute, 11016, Kiryat Shmona, Israel

**Keywords:** Environmental DNA, Equidae, Gastrointestinal nematodes, Reintroduction, Strongylidae

## Abstract

Reintroduced animals face disease risks, potentially impacting both the reintroduced and the local wildlife/domestic populations. This study focuses on the Asiatic wild asses (*Equus hemionus*) reintroduced to the Negev desert in southern Israel. Despite potential threats of disease spill-over to and from domesticated donkeys and horses in the area, there are no records of the gastrointestinal nematodes (GIN) of the wild ass population. We used DNA metabarcoding on fecal samples of wild asses collected across seasons and habitats, near water sources that they frequently use. Ten GIN species were detected in the feces, nine belonging to the family Strongylidae, which commonly infects and causes disease in equids worldwide, such as horses, zebras, and donkeys. Some of these Strongylidae species are also found in domesticated equids in Israel, thus raising concerns regarding potential parasite transmission between wild and domestic animals. The high prevalence of certain GIN species suggests frequent transmission, likely due to the congregation of the wild asses around water sources. While we observed statistically significant variations in some GIN species across seasons and habitats, we did not find clear overall differences between GIN communities. DNA metabarcoding proves to be a valuable tool for identifying GIN species in wild animals, with potential applications in monitoring their health and preventing disease transmission to and from domestic animals.

## Highlights

• A first study to identify parasitic gastrointestinal nematodes (GIN) in the reintroduced Asiatic wild ass in Israel.
• Ten GIN species were found, nine from the Strongylidae family, which are common parasites of equids.
• High prevalence of GIN species suggests frequent parasite transmission probably around water sources.
• DNA metabarcoding of fecal samples is an efficient, non-invasive way to identify GIN in wild animals.

## Graphical abstract

**Figure:**
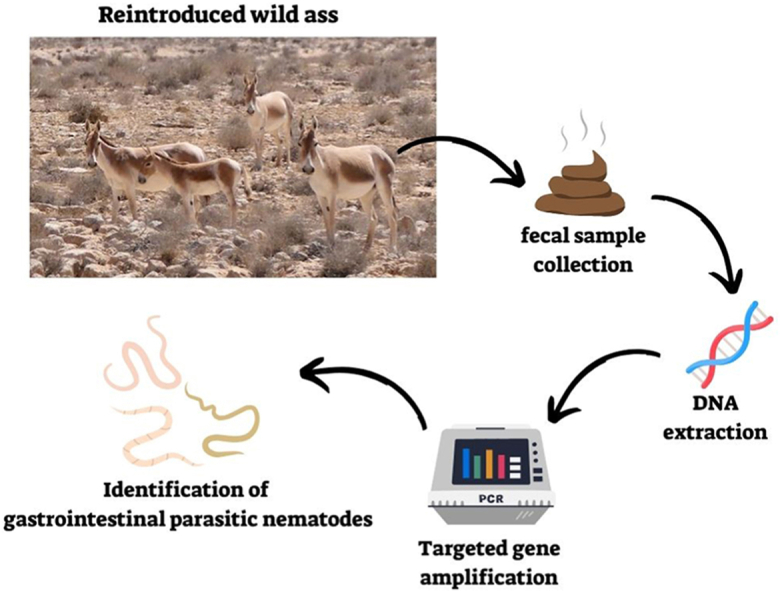

## 1 Introduction

Reintroduction is the intentional movement of an organism into a part of its historical range from which it has become extinct. Reintroduction aims to establish a self-sustaining population of the organism and restore ecological function (Armstrong and Seddon, 2008). Reintroduction programs should include health surveillance since diseases and parasites may threaten their success (Mathews et al., 2006), potentially impacting other wildlife populations through disease spill-over. The risks include high pathogen susceptibility due to reintroduction-related stress, pathogen introduction, and direct transmission through contact with local species (Kock et al., 2010).

The Asiatic wild ass (*Equus hemionus*) was once abundant in deserts and mountain steppes in western Asia. The local Middle Eastern subspecies (*E. h. hemippus*) became extinct in the early twentieth century. In 1968, a breeding core was established in southern Israel (Hai-Bar Yotveta Reserve). That core included six Persian wild asses (*E*. *h*. *onager*; three males, three females) and five Turkmenian wild asses (*E. h. kulan*; two males, three females). The wild asses were first released to the Negev desert (a region in southern Israel) in 1982 (Renan et al., 2018; Saltz and Rubenstein, 1995). The current population is estimated to contain a few hundred individuals spread across the Negev desert (Davidson et al., 2013).

Gastrointestinal nematodes (GIN) are a group of parasites that reside in the digestive system and can cause various gastrointestinal diseases. Many GIN of ungulates have a multi-phase lifecycle, which includes a parasitic phase inside the host and a free-living phase, in which the larvae inhabit the pasture and infect the host through ingestion (Walker and Morgan, 2014). The most common and prominent GIN in equids are the strongyles (family Strongylidae), which are relatively generalist and are capable of infecting different species, including zebras, horses, and donkeys (Abbas et al., 2024; Kuzmina et al., 2020; Tombak et al., 2021). This family can be highly pathogenic, causing gastrointestinal diseases and even death (Lichtenfels et al., 2008; Molla et al., 2015; Tombak et al., 2021).

Since their reintroduced to the Negev over 40 years ago, the wild ass population has grown and expanded, increasing the frequency of interactions with other species in the Negev, including ungulates. Therefore, the potential disease spill-over to domesticated horses and donkeys in the area increases. Additionally, equid-associated GIN (mainly strongyles) were surveyed in domesticated horses from farms across Israel, including a few sites that are inhabited by wild asses in the Negev. The GIN were found in about 25% of all farms surveyed, despite routine deworming regimes (Levy et al., 2015).

In recent years, DNA metabarcoding (amplification and high throughput sequencing of a specific target gene from an environmental sample) has been used to investigate various aspects, including diet (e.g., Kartzinel et al., 2015), cryptic interactions (e.g., Berman and Inbar, 2022), and parasites in a large variety of animals (e.g., Abbas et al., 2024; Avramenko et al., 2015; Davey et al., 2023). It allows the identification of multiple species, regardless of their life stage (Avramenko et al., 2015; Davey et al., 2021; Queiroz et al., 2020). Using DNA metabarcoding, we examined whether we can detect and identify GIN in wild ass feces as potential means for monitoring wildlife health and possible spill-over risks. We addressed the following questions: (1) Which parasitic GIN species can be identified in the reintroduced wild ass population in the Negev? (2) Do the GIN species vary between habitats and seasons?

## 2 Materials and methods

### 2.1 Study area

**Fig. 1:**
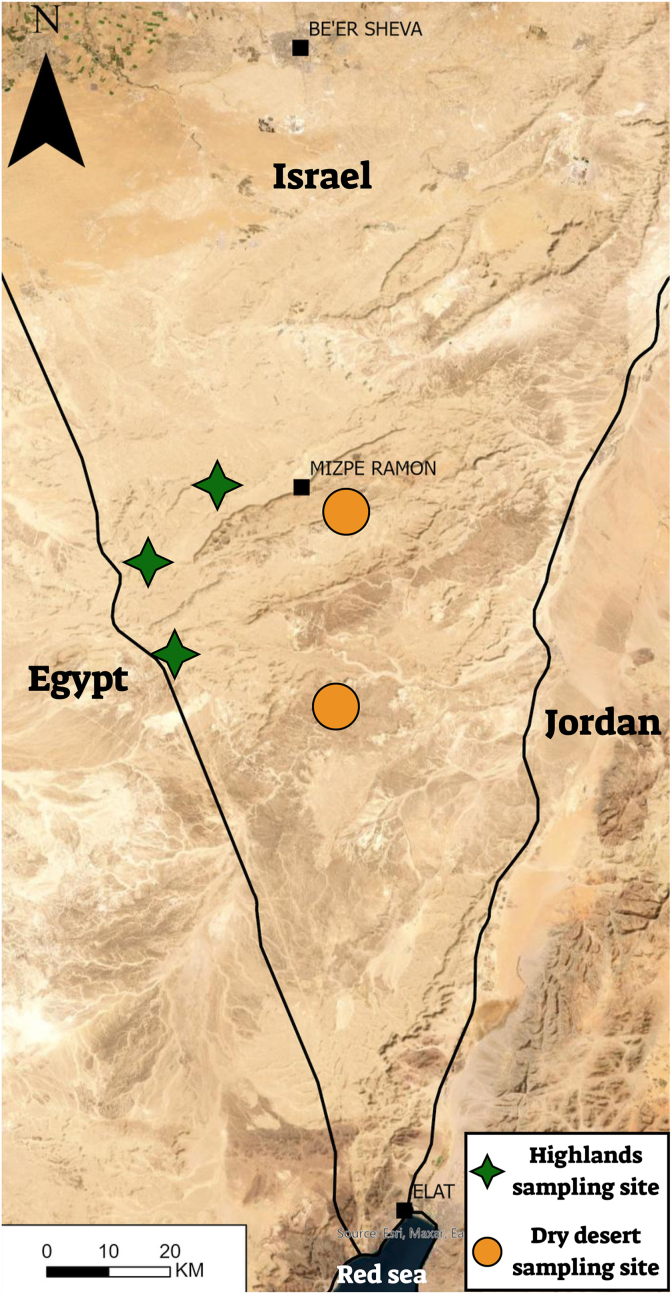
A map of the study area and wild ass fecal sample collection sites - In the Negev highlands and the Negev desert in Israel.

The study was conducted in two habitats in southern Israel - the Negev highland and the dry Negev desert (Fig. 1). The Negev highland is a part of the Irano-Turanian phytogeographical zone, which is characterized by relatively cold winters, hot, dry summers, and dwarf-shrub steppe vegetation with an average of 95 mm annual precipitation (elevation up to 1000 m ABSL). The dry Negev desert is characterized by warmer winters and hot summers with 54 mm annual rainfall on average. The vegetation in this region is mainly restricted to wadis (Renan et al., 2018).

### 2.2 Sample collection

Fecal samples were collected around five artificial water sources established by the Israel National Parks Authority (INPA) – three in the Negev highlands and two in the Negev desert (Fig. 1; INPA collection permit 2022/43127). These water sources may attract numerous (up to hundreds) wild asses providing convenient collection sites for fresh feces from multiple individuals. Samples were collected during summer (June 2022), fall (October 2022), winter (January 2023) and spring (April 2023). Samples were collected at sites at least 5 m apart to avoid sampling the same individual. Each sample contained 50 mL of feces that was kept in a cooler with ice until returning to the laboratory, where it was stored at −80 °C until further processing. In total, 100 fecal samples were analyzed, five samples from each site and season (a total of 60 samples from the Negev highlands and 40 from the Negev desert, Supplementary S1).

### 2.3 DNA extraction and PCR amplification

Before DNA extraction, the fecal samples were thoroughly mixed and homogenized. DNA extraction was performed using the ZymoBiomics miniprep kit (Zymo Research, Catalog Number D4300, Irvine, CA), using the manufacturer protocol. The amplification of GIN DNA was performed using the Gasser et al. (1993) ITS-2 markers (NC1: ACGTCTGGTTCAGGGTTGTT, NC2: TTAGTTTCTTTTCCTCCGCT; 311–331 bp amplicon) which were previously used to identify nematode species from fecal samples of cattle, sheep, bison, and horses (Queiroz et al., 2020). PCR conditions were 95 °C for 5 min, followed by 35 cycles of 95 °C for 30 s, 48 °C for 30 s and 72 °C for 45 s. PCR was completed with a step of 5 min at 72 °C. Negative controls were carried out for each PCR assay and verified by agarose gel electrophoresis. Finally, the PCR products were stored at −20 °C.

### 2.4 Sequencing process

A second PCR amplification was performed in 10 μL reactions in 96-well plates using repliQa HiFi ToughMix. Each well received a primer pair with a unique 10-base barcode from the Access Array Barcode Library for Illumina (Fluidigm, South San Francisco, CA; Item# 100–4876). One microliter of PCR product from the first stage amplification was used as a template for the 2nd stage, without cleanup. Cycling conditions were 98 °C for 2 min, followed by 8 cycles of 98 °C for 10 min, 60 °C for 1 min and 68 °C for 1 min. Libraries were then pooled and sequenced with a 15% phiX spike-in on an Illumina MiSeq sequencer employing V3 chemistry (2x300 base paired-end reads). One sample was removed after this stage due to lacking sequences, retaining 99 samples. Library preparation and sequencing were performed at the Genomics and Microbiome Core Facility (GMCF) at Rush University, Chicago, Illinois, USA.

### 2.5 Sequence analysis and bioinformatics

The dada2 pipeline (Callahan et al., 2016) (dada2 package version 1.26.0) was used for sequence data processing. Sequences were filtered and trimmed for quality using the ‘filterAndTrim’ command with the parameters: maxN set to zero, maxEE set to 3 and truncLen set to 250 bases for both forward and reverse reads. Primer sequences were also trimmed. The sequence error estimation model and error corrections were done using the ‘learnErrors’ and ‘dada’ commands using default parameters. Forward and reverse reads were merged with minimum overlap set at 90 bp. Suspected chimera were detected and removed using the command ‘removeBimeraDenovo’. Following, a count table was produced for the amplicon sequence variants (ASV) in each sample from all seasons. ASV sequences were aligned to the NCBI nt database for taxonomic identification using BLAST. Up to 50 BLAST hits per sequence were used for taxonomic identification using the Lowest Common Ancestor (LCA) in ‘MEGAN community edition’ (v.6.21.11 (Huson et al., 2016);) with parameters set to min score = 100, max expected = 1.0e^-9, top percent = 1. The count table was parsed to retain only ASVs identified as nematodes that were prevalent in more than one sample (Supplementary S2). A total of 3,945,792 reads in 199 nematode ASVs and 99 samples were retained (Supplementary S3).

### 2.6 Data analysis and statistics

ASVs were binned based on their respective GIN up to the species level, as effectively done in previous studies (e.g. Avramenko et al., 2015; Davey et al., 2023; Queiroz et al., 2020). We then examined the prevalence (presence/absence) data of GIN species to understand how their distribution varied across different habitats and seasons. We opted to use prevalence data due to the relatively small sample size. A smaller sample size can limit the reliability of abundance estimates (McMurdie and Holmes, 2014). Therefore, prevalence data may provide more valuable information regarding the distribution of GIN species throughout the year and across the two different habitats. To ensure a more robust understanding, we focused only on prevalent species found in at least ten samples, resulting in a final set of six GIN species (Supplementary S4). To ensure these six species were indeed dominant, we examined the total number of reads of each identified species.

We employed a Fisher's exact test to assess differences in nematode species prevalence across seasons and habitats. We performed pairwise comparisons for seasonal data using Fisher's pairwise exact test whenever the initial test indicated significant differences. We adjusted p-values from the pairwise tests using the Benjamini-Hochberg (FDR) method to account for multiple comparisons.

## 3 Results

**Fig. 2:**
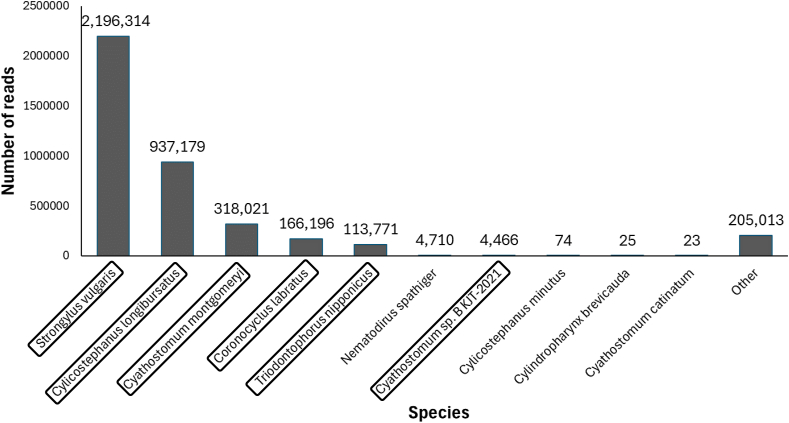
Density plot showing the number of reads of gastrointestinal nematode species identified in wild ass fecal samples collected from two habitats in the Negev highlands and the Negev desert. Highlighted species indicate the species with the highest prevalence, which were used for the final statistical analysis. “Other” includes the 21 ASVs that were only identified to the family or genus level (i.e., no species identification).

DNA metabarcoding analysis of wild asse feces revealed a total of 199 unique ASVs belonging entirely to the order Strongylida. All ASVs were binned to species, apart from 21, which were only identified to the family and genus level. We identified 10 GIN species, nine of them from the family Strongylidae, which are common in equids worldwide, including horses, zebras, and donkeys. One species belonged to the family Molineidae (*Nematodirus spathiger*), a common parasite of ruminants such as sheep and cattle. *Strongylus vulgaris* and *Cylicostephanus lungibursatus* were highly prevalent and appeared in over 80% of all samples (83 and 80 samples, respectively). Six species with a prevalence exceeding 10% in the samples were chosen to further analyze seasonal and habitat variations (Table 1). These six species were not only the most prevalent, but also the most dominant species, exhibiting the highest number of reads across all samples, thus presenting the majority of sequences (Fig. 2).

**Table 1:** **Prevalence (sample count) of identified gastrointestinal nematodes species in wild ass fecal samples.** Highlighted species (prevalence of over ten samples) were used for the statistical analysis.

Family	Species	Prevalence
**Strongylidae**	***Strongylus vulgaris***	**83/99**
**Strongylidae**	***Cylicostephanus longibursatus***	**80/99**
**Strongylidae**	***Cyathostomum montgomeryi***	**62/99**
**Strongylidae**	***Coronocyclus labratus***	**52/99**
**Strongylidae**	***Triodontophorus nipponicus***	**31/99**
**Strongylidae**	***Cyathostomum* sp. *B KJT-2021***	**12/99**
Strongylidae	*Cylicostephanus minutus*	3/99
Strongylidae	*Cyathostomum catinatum*	2/99
Strongylidae	*Cylindropharynx brevicauda*	2/99
Molineidae	*Nematodirus spathiger*	2/99

**Fig. 3:**
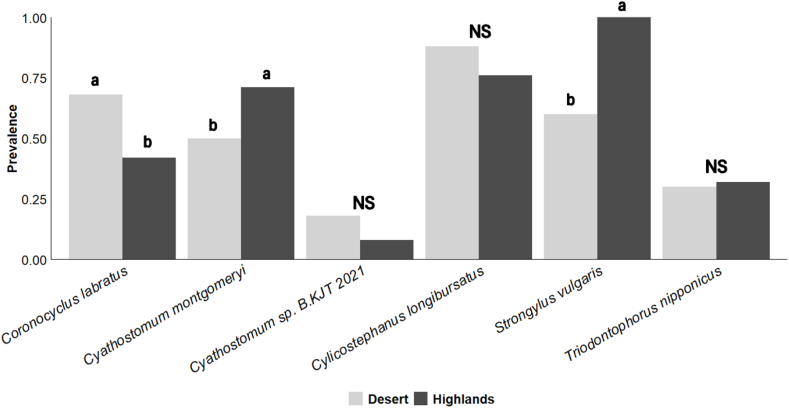
Prevalence (presence/absence) of gastrointestinal nematode species identified in wild ass fecal samples collected from two habitats in the Negev highlands and the Negev desert. Different letters indicate significant differences based on Fisher's exact test.

The six prevalent GIN species were found in all seasons. The prevalence of *Cyathostomum* sp. *B.KJT.2021* (Fisher's exact test, *P* = 0.014) and *Triodontophorus nipponicus*) (Fisher's exact test, *P* < 0.001) was significantly higher in summer compared to winter samples. The prevalence of *Triodontophorus nipponicus* was significantly higher during winter and spring compared to summer and fall. Similarly, all six prevalent species appeared both in the samples collected from the Negev highlands and the Negev desert. *Cyathostomum montgomeryi* (Fisher's exact test, *P* = 0.037) and *Strongylus vulgaris* (Fisher's exact test, *P* < 0.001) were significantly more prevalent in the Negev highlands, while *Coronocyclus labratus* (Fisher's exact test, *P* = 0.023) was more prevalent in the Negev desert (Fig. 3).

## 4 Discussion

Our study demonstrates that DNA metabarcoding of feces is an efficient, non-invasive tool for identifying GIN of wild Equids. In this study, we provide the first record of GIN in reintroduced wild asses in Israel, all belonging to the Strongylida order. We identified, for the first time in Israel, GIN species from the genera *Coronocyclus, Cyathostomum, Cylindropharynx,* and *Cylicostephanus*, which appeared in over 10% of the wild ass fecal samples. The relatively low number of GIN species we identified in the wild ass population (ten species) might be due to a bottlenecked parasite community, caused by a small breeding core (Poissant et al., 2021). Additionally, the high temperatures and solar radiation in the Negev may contribute to reduced GIN egg and larvae viability (Poissant et al., 2021) resulting in a small number of identified GIN.

Feces were collected from artificial water sources provided to the wild asses by the INPA. Given their high dependence on water (Giotto et al., 2015), these sources are dense gathering sites with frequent (daily) visits by many individuals. Since strongylid infections occur through larval ingestion from contaminated environments (Durette-Desset et al., 1994; Sharir et al., 1987), these congregations likely facilitate higher parasite transmission. This may explain the high prevalence of GIN observed, particularly species like *S*.*vulgaris* and *C*.*lungibursatus*, which appeared in many samples (Table 1). Our findings suggest widespread infection within the wild ass population due to the frequent intermingling at and around water sources.

We identified six GIN species belonging to four genera that have not been previously recorded in equids in Israel to the best of our knowledge. All these species are classified as Cyathostomins, or small strongyles (Corning, 2009). *Cylicostephanus longibursatus,* which was highly prevalent in the wild ass feces (Table 1), together with *Cylicostephanus minutus, Cyathostomum catinatum,* and *Coronocyclus labratus,* are common horse parasites (Corning, 2009; Morariu et al., 2016; Silva et al., 1999), raising the possibility of transmission between wild asses and nearby domesticated horses and donkeys. Furthermore, we found *Cylindropharynx brevicauda* and *Cyathostomum montgomeryi*, species known to infect African donkeys and zebras but rarely reported in domestic horses (Eysker and Pandey, 1989; Kharchenko et al., 2009).

*Nematodirus spathiger* was found in only two fecal samples (Table 1) with a relatively low number of reads (Fig. 2; 0.12% of all reads). This GIN is a common parasite of various mammalian herbivores, including ruminants such as sheep and cattle, llamas, and even rabbits (Audebert et al., 2004; Oliver et al., 2014; Petrigh and Fugassa, 2014), but it does infect equids. This parasite was probably coincidentally transmitted to wild ass from pastures contaminated by different ruminants (such as gazelles), especially near water sources (see Webster and Mackay, 1969).

Intriguingly, *S. vulgaris* was highly prevalent in the wild ass feces (Table 1). When not treated with anthelmintics, *S. vulgaris* is known to be highly detrimental in domesticated horses, causing severe intestine damage, peritonitis, colic, and even death (Nielsen et al., 2021). However, *S. vulgaris* appears to have minimal impact on the health of these wild asses (note that our analysis is qualitative and not quantitative), even without any deworming interventions. This observation might be attributed to an increased tolerance developed by wild equids to parasites due to their long history of exposure (Tombak and Rubenstein, 2023). While domesticated horses are usually treated against *S. vulgaris*, it is prevalent in wild equids, which can serve as a reservoir of this GIN (Cain et al., 2018; Harvey et al., 2019).

Variations in the prevalence of some GIN species were visible across seasons and habitats (Fig. 3), but these variations did not clearly distinguish between the total GIN communities in the Negev highlands and the Negev desert, nor between seasons. Nevertheless, identifying trends at these scales requires a larger sample size, more study sites, and tracking over the years. Additionally, DNA metabarcoding does not provide quantitative information on parasite abundance. Future quantitative research methods, such as fecal egg counts, could provide valuable insights into the potential health impacts of these parasites.

In recent decades, there has been an increase in emerging infectious diseases attributed to the exposure of humans and livestock to pathogens from wildlife. This led to the “One Health” approach, which acknowledges the interconnectedness of humans, domesticated and wild animals, and ecosystem health (Destoumieux-Garzón et al., 2018; Mackenzie and Jeggo, 2019). Nearly all GIN species identified in this study (Strongylidae) commonly infects equids, including horses, zebras, and donkeys (Abbas et al., 2024; Levy et al., 2015; Tombak et al., 2021) and are also found in reintroduced wild asses in Kazakhstan (Gliga et al., 2020). Translocation and reintroduction projects can insert pathogens into the release sites. For example, African horse sickness was brought to Spain by two translocated zebras and *Plasmodium* spp carried by wild turkeys to North America (Kock et al., 2010). In many parts of the Negev (especially in the highlands), there is a distribution overlap between wild asses and domesticated horses and donkeys that are used for recreational activities or by local Bedouin communities. The presence of domestic animals near the reintroduced wild asses increases the potential reciprocal parasite transmission between domestic equids and wild asses (Walker and Morgan, 2014). Notably, *S. vulgaris* and a species of *Triodontophorus* were identified in both wild asses and horses in Israel (Levy et al., 2015; Sharir et al., 1987).

In conclusion, we used DNA metabarcoding to identify GIN in wild ass feces from the Negev highlands and Negev desert. Since large-scale parasite monitoring of GIN communities in wild populations is challenging (Davey et al., 2023), DNA metabarcoding can be a useful tool for identifying and monitoring GIN in wild animals as it does not require any direct contact with the animals and can be easily repeated for long-term monitoring. Identifying Strongylids in the wild asses in the Negev highlights the importance of “One Health” considerations during reintroductions. Gaining a full view of the parasitology of the reintroduced wild asses can help develop strategies to minimize parasite transmission and ensure the health of wild asses and domesticated equids in the area.

## Funding

This research received no specific grant from funding agencies in the public, commercial, or not-for-profit sectors.

## Data availability statement

Data available from the National Center for Biotechnology Information (NCBI) GenBank: Accession number PRJNA1119080.

## CRediT authorship contribution statement

**R. Forman:** Writing – review & editing, Methodology, Formal analysis, Conceptualization. **M. Lalzar:** Writing – review & editing, Formal analysis. **M. Inbar:** Writing – review & editing, Resources, Methodology, Conceptualization. **T.S. Berman:** Writing – review & editing, Methodology, Formal analysis, Conceptualization.

## Declaration of competing interest

The authors declare that the research was conducted in the absence of any commercial or financial relationships that could be construed as a potential conflict of interest.
